# Knockdown of hepatocyte Perilipin-3 mitigates hepatic steatosis and steatohepatitis caused by hepatocyte CGI-58 deletion in mice

**DOI:** 10.1093/jmcb/mjac055

**Published:** 2022-09-15

**Authors:** Xinyu Bao, Xiaogen Ma, Rongfeng Huang, Jianghui Chen, Haoran Xin, Meiyu Zhou, Lihua Li, Shifei Tong, Qian Zhang, Guanghou Shui, Fang Deng, Liqing Yu, Min-Dian Li, Zhihui Zhang

**Affiliations:** Department of Cardiovascular Medicine, Center for Circadian Metabolism and Cardiovascular Disease, Southwest Hospital, Army Medical University, Chongqing 400038, China; Department of Cardiovascular Medicine, Center for Circadian Metabolism and Cardiovascular Disease, Southwest Hospital, Army Medical University, Chongqing 400038, China; Department of Cardiovascular Medicine, Center for Circadian Metabolism and Cardiovascular Disease, Southwest Hospital, Army Medical University, Chongqing 400038, China; Department of Cardiovascular Medicine, Center for Circadian Metabolism and Cardiovascular Disease, Southwest Hospital, Army Medical University, Chongqing 400038, China; Department of Cardiology, The Third Affiliated Hospital of Chongqing Medical University, Chongqing 401120, China; Department of Cardiovascular Medicine, Center for Circadian Metabolism and Cardiovascular Disease, Southwest Hospital, Army Medical University, Chongqing 400038, China; Department of Cardiovascular Medicine, Center for Circadian Metabolism and Cardiovascular Disease, Southwest Hospital, Army Medical University, Chongqing 400038, China; Department of Cardiovascular Medicine, Center for Circadian Metabolism and Cardiovascular Disease, Southwest Hospital, Army Medical University, Chongqing 400038, China; Department of Cardiology, The Third Affiliated Hospital of Chongqing Medical University, Chongqing 401120, China; Department of General Medicine, Southwest Hospital, Army Medical University, Chongqing 400038, China; State Key Laboratory of Molecular Developmental Biology, Institute of Genetics and Developmental Biology, Chinese Academy of Sciences, Beijing 100101, China; Department of Pathophysiology, College of High Altitude Military Medicine, Army Medical University, Chongqing 400038, China; Key Laboratory of Extreme Environmental Medicine, Ministry of Education of China, Chongqing 400038, China; Key Laboratory of High Altitude Medicine, PLA, Chongqing 400038, China; Division of Endocrinology, Diabetes and Nutrition, Department of Medicine, University of Maryland School of Medicine, Baltimore, MD 21201, USA; Department of Cardiovascular Medicine, Center for Circadian Metabolism and Cardiovascular Disease, Southwest Hospital, Army Medical University, Chongqing 400038, China; Department of Cardiovascular Medicine, Center for Circadian Metabolism and Cardiovascular Disease, Southwest Hospital, Army Medical University, Chongqing 400038, China

**Keywords:** lipid droplet, lipolysis, rare human disease, nonalcoholic fatty liver disease, nonalcoholic steatohepatitis, Chanarin–Dorfman syndrome

## Abstract

Comparative gene identification-58 (CGI-58), also known as α/β hydrolase domain containing 5, is the co-activator of adipose triglyceride lipase that hydrolyzes triglycerides stored in the cytosolic lipid droplets. Mutations in *CGI-58* gene cause Chanarin–Dorfman syndrome (CDS), an autosomal recessive neutral lipid storage disease with ichthyosis. The liver pathology of CDS manifests as steatosis and steatohepatitis, which currently has no effective treatments. Perilipin-3 (Plin3) is a member of the Perilipin–ADRP–TIP47 protein family that is essential for lipid droplet biogenesis. The objective of this study was to test a hypothesis that deletion of a major lipid droplet protein alleviates fatty liver pathogenesis caused by CGI-58 deficiency in hepatocytes. Adult CGI-58-floxed mice were injected with adeno-associated vectors simultaneously expressing the Cre recombinase and microRNA against Plin3 under the control of a hepatocyte-specific promoter, followed by high-fat diet feeding for 6 weeks. Liver and blood samples were then collected from these animals for histological and biochemical analysis. Plin3 knockdown in hepatocytes prevented steatosis, steatohepatitis, and necroptosis caused by hepatocyte CGI-58 deficiency. Our work is the first to show that inhibiting Plin3 in hepatocytes is sufficient to mitigate hepatocyte CGI-58 deficiency-induced hepatic steatosis and steatohepatitis in mice.

## Introduction

Chanarin–Dorfman syndrome (CDS) is a neutral lipid storage disease with ichthyosis caused by homozygous mutations in α/β hydrolase domain containing 5 (ABHD5) gene, also known as comparative gene identification-58 (CGI-58) (Schweiger et al., 2009; Grabner et al., 2021). Patients with CDS accumulate lipid droplets in almost all cell types, including hepatocytes, leading to hepatomegaly and steatohepatitis (Cakmak and Bagci, 2021). It has been well established that CGI-58 functions as the co-activator of adipose triglyceride lipase (ATGL), promoting lipid droplet lipolysis, i.e. hydrolysis of triglycerides stored in the cytosolic lipid droplets (Grabner et al., 2021), though it has ATGL-independent functions (Zhang et al., 2014; Jebessa et al., 2019; Yu et al., 2020). Liver-specific inhibition of CGI-58 is sufficient to induce hepatomegaly, hepatic steatosis, and steatohepatitis in mice (Brown et al., 2010; Sun and Lazar, 2013), indicating that the liver pathologies seen in CDS patients directly originate from CGI-58 deficiency in the liver.

Lipid droplet is a phospholipid monolayer organelle that stores cellular energy as neutral lipids, such as triglycerides and cholesterol esters (Olzmann and Carvalho, 2019; Seebacher et al., 2020; Mashek, 2021). The Perilipin–ADRP–TIP47 (PAT) protein family has five members named Perilipin-1 (Plin1), Plin2, Plin3, Plin4, and Plin5, all of which are lipid droplet coat proteins (Kimmel and Sztalryd, 2016). Overall, perilipins likely stabilize lipid droplets by preventing CGI-58 to activate ATGL for activation of lipid droplet lipolysis (Wolins et al., 2006; Sanders et al., 2015; Gallardo-Montejano et al., 2016; Seibert et al., 2020). PLIN2, PLIN3, and PLIN5 are the predominant isoforms in the hepatocytes, whereas PLIN1 and PLIN4 are sparsely present (Kimmel and Sztalryd, 2016). Levels of Plin2 and Plin3 are elevated in human and mouse fatty livers (Pawella et al., 2014; Kimmel and Sztalryd, 2016; Mashek, 2021). Liver Plin2 deficiency decreases the hepatic triglyceride content by at least 60% in diet-induced obese mice (Chang et al., 2006; Varela et al., 2008; Tsai et al., 2017). Depletion of hepatic Plin3 by antisense oligonucleotides reduces hepatic steatosis by 35%–52% in the mouse liver (Carr et al., 2012). Considering the role of perilipins in stabilizing the lipid droplet structure, we hypothesized that deletion of the lipid droplet coat proteins may inhibit lipid droplet biogenesis, thereby preventing intracellular lipid droplet deposition and associated lipotoxicity.

In this study, we specifically tested whether silencing of hepatocyte Plin3 attenuates hepatic steatosis and steatohepatitis caused by hepatocyte-specific knockout of *CGI-58* gene. We deleted hepatocyte CGI-58 and silenced Plin3 simultaneously in adult mice by introducing Cre recombinase and microRNA (miRNA) against Plin3 under the control of hepatocyte-specific promoter into the CGI-58-floxed mice. The animals were then fed a high-fat diet (HFD) for 6 weeks, followed by the analysis of liver pathohistology as well as liver and blood biochemistry. We found that Plin3 knockdown in hepatocytes reduced hepatic steatosis, steatohepatitis, and necroptosis caused by hepatocyte CGI-58 deficiency in mice.

## Results

### Silencing of Plin3 in hepatocytes decreases lipid accumulation in CGI-58-deficient mice fed a normal chow diet

**Figure 1:**
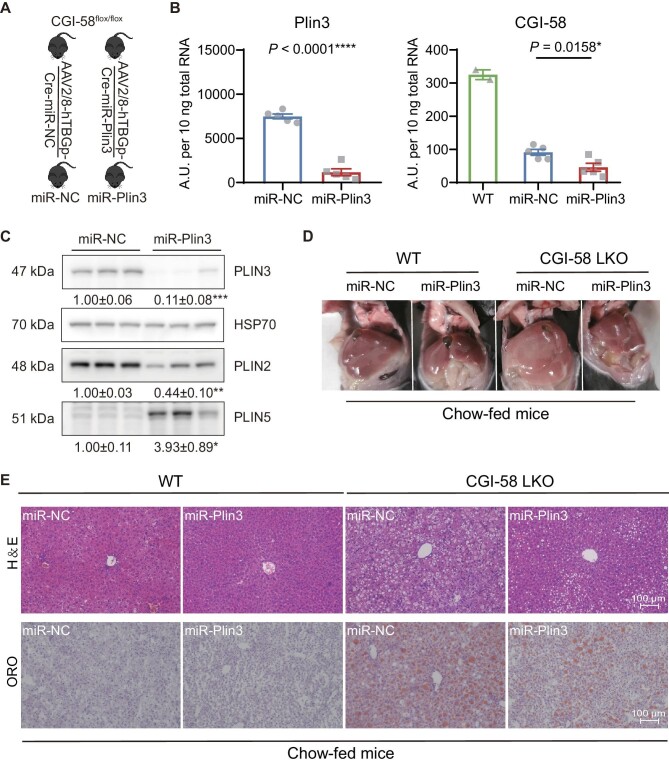
Silencing of Plin3 in hepatocytes decreases lipid accumulation caused by hepatocyte CGI-58 deficiency in mice fed a regular chow diet. (**A**) Scheme for hepatocyte-specific deletion of *CGI-58* gene with or without hepatocyte-specific knockdown of Plin3 in female mice. (**B**) Hepatic mRNA expression levels of Plin3 and CGI-58 (*n* = 5). Two wild-type (WT) C57BL/6J mouse livers were used as a reference to obtain the baseline level of *CGI-58* transcript. (**C**) Western blots of major perilipin proteins and HSP70 in mouse livers. (**D**) Gross appearance of livers. (**E**) H&E and Oil Red O staining of liver sections. CGI-58 LKO, homozygous CGI-58-floxed mice transduced with TBG-Cre AAVs; miR-NC, control miRNA coexpressed with TBG-Cre; miR-Plin3, Plin3 miRNA coexpressed with TBG-Cre. Data are presented as mean ± SEM, *t*-test, *P < 0.05, **P < 0.01, ***P < 0.001, ****P < 0.0001.

To test whether targeting hepatic PAT proteins would improve fatty liver development and progression caused by CGI-58 deficiency, we transduced CGI-58^flox/flox^ mice with adeno-associated vectors (AAVs) expressing the Cre recombinase transgene and miRNA against *Plin3* (miR-Plin3) or a control miRNA sequence (miR-NC) under the hepatocyte-specific promoter derived from human thyroxine binding globulin (hTBG) gene (Figure 1A). Gene expression analysis of liver samples obtained from the HFD-fed mice showed that miR-Plin3 knocked down hepatic *Plin3* transcript by >84% (Figure 1B) and had no effects on the hepatic mRNA levels of Plin2 and Plin5 (Supplementary Figure S1A). Expression of Cre decreased the *CGI-58* transcript by 72%–86% in the liver (Figure 1B).

We further examined the protein levels of perilipins and CGI-58 in the liver tissues. miR-Plin3 reduced PLIN3 and PLIN2 proteins by 89% and 56%, respectively, while increasing PLIN5 by 3.9-fold (Figure 1C). Hepatic CGI-58 level was ablated to the undetectable level in the AAV-Cre-transduced liver (Supplementary Figure S1B). Thus, we successfully generated a mouse model that is deficient in CGI-58 with or without PLIN3 in the hepatocytes.

Liver CGI-58 knockout mice are known to develop hepatic steatosis even under normal chow diets (Guo et al., 2013). We assessed hepatic lipid accumulation in the 11-week-old chow-fed female mice. As expected, hepatocyte-specific deletion of CGI-58 caused hepatic steatosis in these animals as evidenced by gross appearance of the livers, hematoxylin–eosin (H&E) staining, and Oil red O staining (Figure 1D and E). Knockdown of hepatocyte Plin3 clearly alleviated hepatocyte CGI-58 deficiency-induced hepatic steatosis (Figure 1E).

### Silencing of hepatocyte Plin3 prevents hepatic steatosis in hepatocyte CGI-58-deficient mice fed a HFD

To obtain the basic metabolic parameters of the mice, we monitored body weight changes for up to 6 weeks but did not observe any significant differences between the two groups (Supplementary Figure S1C). Glucose tolerance test (GTT; Supplementary Figure S1D) and insulin tolerance test (ITT; Supplementary Figure S1E) did not show any differences between miR-Plin3 and miR-NC groups (Supplementary Table S2). The mass of perigonadal white adipose tissue (pgWAT), as well as the size distribution of adipocytes in pgWAT, was not altered by miR-Plin3 treatment (Supplementary Figure S2A and B). Also, mRNA levels of perilipins in pgWAT were similar between the two groups (Supplementary Figure S2C), confirming hepatocyte-specific Plin3 knockdown.

**Figure 2:**
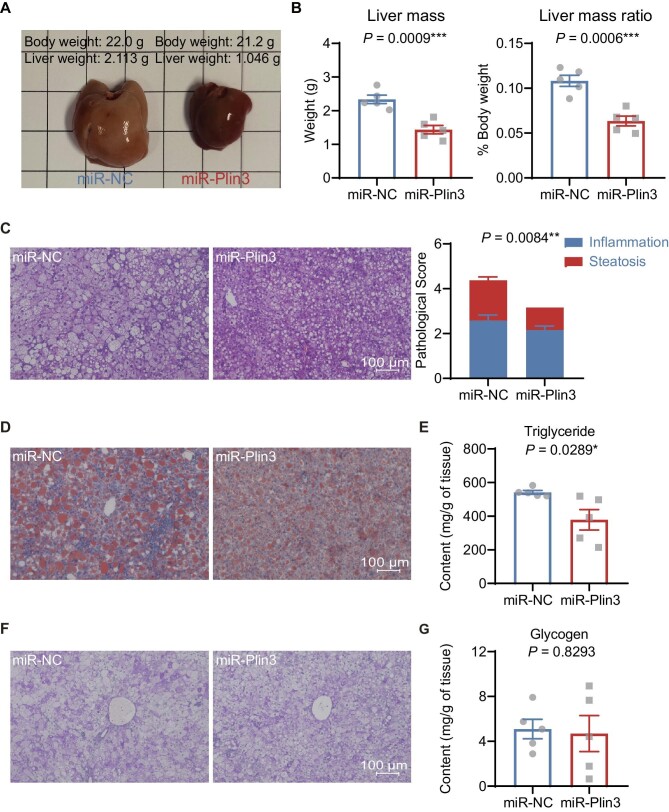
Plin3 knockdown prevents hepatic steatosis in CGI-58-deficient mice fed a HFD. (**A**) Liver morphology. (**B**) The mass and mass ratio of liver tissues (*n* = 5). (**C**–**G**) Pathological and biochemical examination in the CGI-58^Δliver^ livers expressing miR-NC or miR-Plin3 (*n* = 5). (**C**) Histology. (**D**) Oil red O staining. (**E**) Triglyceride content. (**F**) Glycogen accumulation detected by PAS staining. (**G**) Glycogen content. Scale bar, 100 μm. Data are presented as mean ± SEM, *t*-test, *P < 0.05, **P < 0.01, ***P < 0.001.

Remarkably, miR-Plin3 treatment improved the gross appearance and significantly decreased the size and mass of livers caused by hepatocyte CGI-58 deletion in mice fed the HFD (Figure 2A and B). CGI-58 deficiency and HFD feeding are known to increase hepatic fat deposition causing hepatomegaly and the formation of yellow liver color. miR-Plin3 may prevent these pathologies by reducing fat accumulation in the liver. Indeed, H&E staining of liver specimens showed that Plin3 knockdown decreased the area of lipid droplets (Figure 2C). Oil red O staining further confirmed the suppression of lipid droplet formation by miR-Plin3 (Figure 2D). Biochemical analysis revealed that Plin3 knockdown significantly reduced the hepatic content of triglycerides from 541.40 ± 11.18 mg/g to 378.40 ± 60.28 mg/g (Figure 2E). Nevertheless, miR-Plin3 did not alter the hepatic content of free fatty acids (Supplementary Figure S3A, 2.62 ± 0.26 μmol/g in miR-Plin3 vs. 2.56 ± 0.15 μmol/g in miR-NC, *P* > 0.05). miR-Plin3 showed a trend to decrease the hepatic content of total cholesterol (Supplementary Figure S3B). There was no significant difference between miR-Plin3 and miR-NC groups in the hepatic glycogen content (Figure 2F and G). Next, we examined the hepatic contents of other macromolecules and found that miR-Plin3 did not significantly alter the contents of DNA and proteins in the liver (Supplementary Figure S3C and D). Overall, the results demonstrate that Plin3 knockdown prevented hepatic steatosis in the HFD-fed hepatocyte CGI-58-deficient mice.

### Silencing of hepatocyte Plin3 prevents steatohepatitis in hepatocyte CGI-58-deficient mice

**Figure 3:**
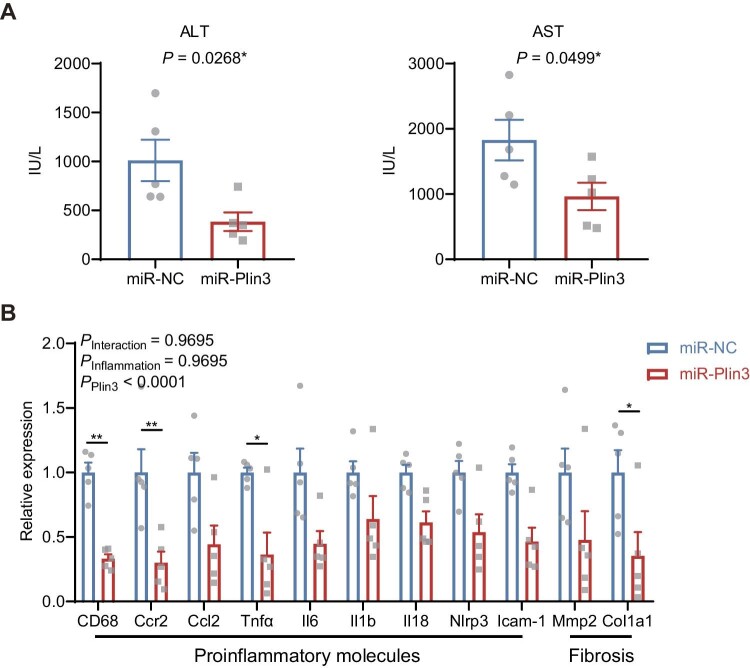
Plin3 knockdown prevents hepatitis in the CGI-58-deficient mice. (**A**) ALT and AST in the serum (*n* = 5). (**B**) Expression of genes involved in inflammation and fibrosis in the CGI-58^Δliver^ livers expressing miR-NC or miR-Plin3 (*n* = 5). Data are presented as mean ± SEM, *t*-test or two-way ANOVA post-hoc Bonferroni tests, *P < 0.05, **P < 0.01.

To examine the overall health of the animals, we performed blood chemistry tests and found that miR-Plin3 treatment significantly increased albumin whereas decreased globulin and total cholesterol (Supplementary Table S2). Remarkably, miR-Plin3 decreased serum levels of alanine aminotransferase (ALT) and aspartate aminotransferase (AST) by >50%, suggesting reduced liver injury and improved liver functions in mice with hepatocyte-specific deficiency of both CGI-58 and Plin3 (Figure 3A). In addition, we observed that the blood biochemical changes were associated with decreased hepatic mRNA levels of proinflammatory cytokine and fibrotic genes, such as *CD68, Ccr2, Tnf*α, and *Col1a1* (Figure 3B). Consistently, pathohistological analysis showed that miR-Plin3 treatment reduced the area of lobular inflammation in the hepatocyte CGI-58-deficient liver (Figure 2C). Collectively, these findings indicate that silencing of hepatocyte Plin3 prevents injury and steatohepatitis caused by hepatocyte CGI-58 deficiency in the mouse liver.

### Plin3 knockdown reduces lipid droplet biogenesis in AML-12 hepatocytes

Plin3 is a lipid droplet protein critically implicated in the biogenesis and stability of lipid droplets. Plin3 knockdown likely hinders efficient formation and/or stability of intracellular lipid droplets, alleviating steatosis. We sought to define how Plin3 regulates dynamics of intracellular lipid droplets at multiple levels. We measured hepatic levels of mRNAs encoding major proteins for fatty acid uptake including CD36 and Fatp1; interestingly, they were suppressed by miR-Plin3 (Supplementary Figure S4A). Western blotting analysis showed that miR-Plin3 modestly reduced protein levels of HSL and pS660-HSL by 50% and 33%, respectively, in the liver (Supplementary Figure S4B and C).

**Figure 4:**
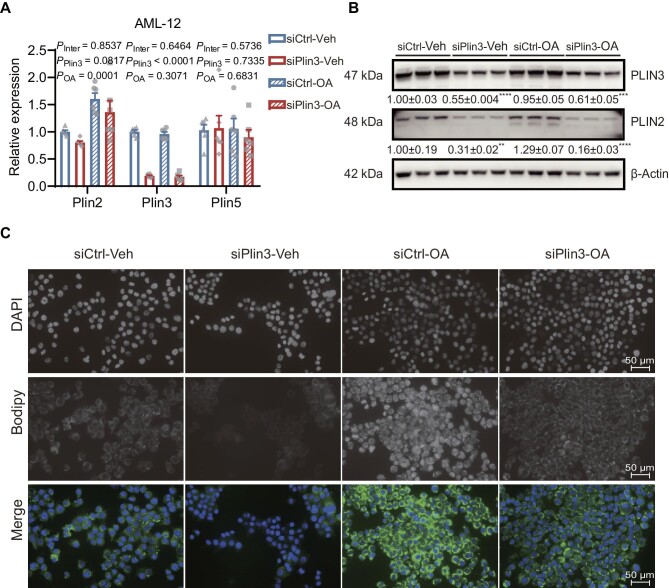
Plin3 knockdown reduces lipid droplet formation in AML-12 hepatocytes. (**A**) Gene expression in AML-12 cells challenged by oleic acid (OA) for 12 h (*n* = 4). Veh, vehicle; siCtrl, control siRNA. (**B**) Western blotting analysis in AML-12 cells (*n* = 3). (**C**) Bodipy staining. Scale bar, 50 μm. Two-way ANOVA post-hoc Bonferroni tests, **P < 0.01, ***P < 0.001, ****P < 0.0001 (siPlin3 vs. siCtrl).

We also studied the cell autonomous effects of Plin3 knockdown on lipid droplet accumulation in AML-12 hepatocytes. Oleic acid treatment increased transcript level of Plin2, but not those of Plin3 and Plin5 in AML-12 hepatocytes (Figure 4A). Small interfering RNA (siRNA) against *Plin3* (siPlin3) significantly decreased Plin3 mRNA level by >80% without altering mRNA levels of Plin2 and Plin5 (Figure 4A). Western blotting analysis confirmed that siPlin3 reduced protein levels of PLIN3 and PLIN2 (Figure 4B). We assessed lipid droplets by the lipophilic neutral fluorophore Bodipy and found that Plin3 knockdown reduced lipid droplet deposition in hepatocytes (Figure 4C). These findings demonstrate that Plin3 is essential for lipid droplet biogenesis in hepatocytes.

Excessive accumulation of intracellular lipid droplet may impair mitochondrial biogenesis and functions. On the other hand, improved mitochondrial biogenesis and functions may counter lipid droplet deposition by oxidizing more fats. To determine whether miR-Plin3 has any effects in this regard, we assessed the mitochondrial copy number (Supplementary Figure S5A) and the abundance of Cps1 and mitochondrial DNA-encoded transcripts in the liver (Supplementary Figure S5B). miR-Plin3 did not significantly alter these parameters, implying that miR-Plin3 treatment does not have a significant impact on the mitochondrial biogenesis and functions in the hepatocyte CGI-58-deficient liver. Therefore, Plin3 knockdown may alleviate steatohepatitis by decreasing lipid droplet biogenesis and fatty acid uptake.

### Silencing of Plin3 in hepatocytes suppresses hepatic necroptosis in hepatocyte CGI-58-deficient mice

**Figure 5:**
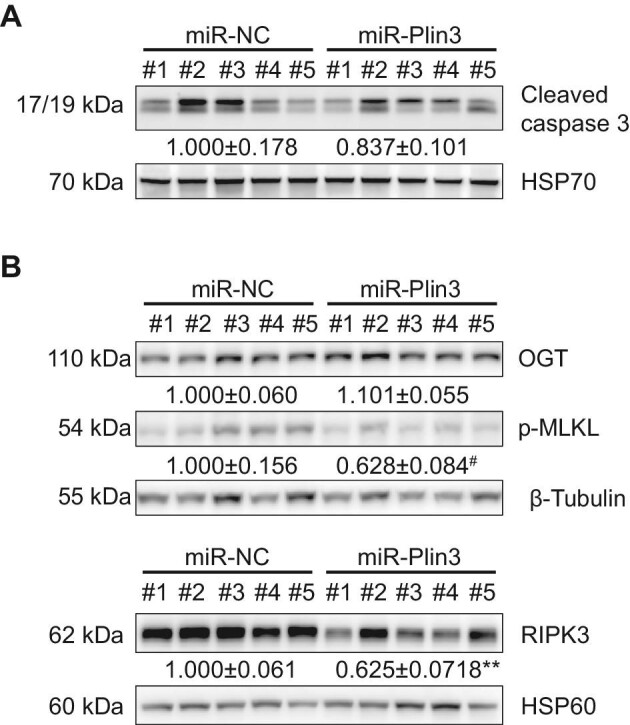
Plin3 knockdown suppresses necroptosis in the CGI-58-deficient liver. Western blotting analysis of markers and regulators involved in apoptosis (**A**) and necroptosis (**B**) in mouse livers. Data are presented as mean ± SEM, *t*-test, ^#^0.05 < *P *< 0.1, **P < 0.01.

Since miR-Plin3 alleviated liver injury and decreased expression levels of inflammation markers, we further examined the differences in cell death pathways between the two groups. We assessed hepatic levels of proteins and protein phosphorylation involved in either apoptosis or necroptosis in hepatocyte CGI-58-deficient mice with miR-Plin3 or control miR-NC. The results showed that miR-Plin3 did not alter the level of cleaved caspase 3 (Figure 5A). Instead, silencing of Plin3 in hepatocytes significantly decreased the levels of MLKL phosphorylation (0.628 ± 0.084 vs. 1.000 ± 0.156, 0.05 < *P* < 0.1) and RIPK3 (0.625 ± 0.071 vs. 1.000 ± 0.061, *P* < 0.01) in the liver (Figure 5B). These results suggest that knockdown of Plin3 in hepatocytes suppresses liver necroptosis in hepatocyte CGI-58-deficient mice.

## Discussion

A major finding of this study is that inhibition of a single member of the PAT protein family is sufficient to mitigate hepatic steatosis and steatohepatitis associated with hepatocyte CGI-58 deficiency and overnutrition. This inhibition does not seem to have adverse impacts on animal health (Supplementary Figure S2). CGI-58 is implicated in the pathogenesis of fatty liver disease through its interactions with other proteins such as PAT proteins and PNPLA family proteins. Mutations in PNPLA3 are linked to human fatty liver disease in all populations (Romeo et al., 2008; Jonas and Schürmann, 2021). CGI-58 was shown to interact with PNPLA3 to regulate hepatic lipid droplet formation (Wang et al., 2019). Therefore, our genetic model is relevant to human fatty liver pathogenesis and is not just a model of a rare genetic disease.

A hallmark of fatty liver disease is the excessive deposition of lipid droplets in the liver (Samuel and Shulman, 2019; Seebacher et al., 2020; Lazarus et al., 2021; Mashek, 2021). Lipid droplet formation and degradation are tightly regulated by numerous lipids and proteins including perilipins (Grabner et al., 2021; Tilg et al., 2021; Mejhert et al., 2022). These lipid droplet coat proteins can stabilize lipid droplets and regulate droplet dynamics. Lipid droplet biology is emerging as a crucial determinant in the pathogenesis of NAFLD (Mashek, 2021). Theoretically, silencing these coat proteins has the potential to prevent lipid droplet formation, thereby mitigating lipid deposition and associated lipotoxicity. Our study is the first to demonstrate that silencing a single lipid droplet coat protein PLIN3 can substantially attenuate hepatic steatosis and steatohepatitis in a murine model of advanced fatty liver disease. The short regimen of HFD feeding does not provide a time window to evaluate hepatic fibrosis and cirrhosis in hepatocyte CGI-58-deficient mice, which should be addressed in a longer regimen. Our findings provide the first proof-of-principle evidence in support of our hypothesis that targeting lipid droplet formation, the PAT family lipid droplet coat proteins in particular, is sufficient to prevent fatty liver and nonalcoholic steatohepatitis (NASH) associated with hepatocyte CGI-58 deficiency and overnutrition.

In hepatocytes, PLIN2 and PLIN3 are the major PAT protein family members that promote lipid storage, knockdown of each of which by antisense oligonucleotides can reduce diet-induced hepatic steatosis (Varela et al., 2008; Carr et al., 2012). Impaired mitochondrial biogenesis is involved in the pathogenesis of fatty liver disease (Sun and Lazar, 2013; Samuel and Shulman, 2019). However, results from our studies on mitochondrial number and functions did not support that miR-Plin3 reduces lipid droplet accumulation in the liver by increasing mitochondrial biogenesis (Supplementary Figure S4B and C). Our results are consistent with the classical role of PLIN3 in supporting lipid droplet formation.

Programmed cell death through necroptosis is emerging as a major driver underneath the pathogenesis of liver injury and fibrosis (Linkermann and Green, 2014; Zhang et al., 2019). Upon excessive tumor necrosis factor or reactive oxygen species, receptor-interacting serine–threonine kinase 3 (RIPK3, also known as RIP3) is activated and phosphorylates MLKL to promote necroptosis (He et al., 2009; Zhang et al., 2009; Sun et al., 2012). It has been recently reported that RIPK3-controlled necroptosis is involved in the pathogenesis of alcoholic steatohepatitis and a spontaneous NASH-like syndrome due to loss of hepatic O-GlcNAc transferase (OGT) (Zhang et al., 2019). We did not detect changes in OGT protein levels in the liver of hepatocyte CGI-58-deficient mice with hepatocyte-specific silencing of Plin3 (Figure 4B). Interestingly, miR-Plin3 decreased necroptosis but not apoptosis in the liver of hepatocyte CGI-58-deficient mice (Figure 4). It remains unknown how PLIN3 differentially regulates these two cell death pathways under our experimental conditions.

In summary, the findings from this study identify hepatocyte PLIN3 as a protein essential for hepatic steatosis and NASH caused by hepatocyte CGI-58 deficiency in HFD-fed mice. Inhibiting proteins involved in the formation of lipid droplets may prevent fatty liver development and progression.

## Materials and methods

### Animal studies

Animal experiments were approved by the Laboratory Animal Welfare and Ethics Committee of Army Medical University (AMU), China. All experiments conform to the relevant regulatory standards of AMU. CGI-58^flox/flox^ mice had the exon 3 of *CGI-58/Abhd5* gene flanked by two loxP sites (Guo et al., 2013). Female CGI-58^flox/flox^ mice were bred by Cyagen Biosciences at the age of 4 weeks, raised in the specific pathogen-free barrier facility of AMU, and acclimated to a 12-h light:12-h dark cycle (light-on at 8 am, light-off at 8 pm) with normal chow food and water ad libitum for two weeks. Animals were assigned to two groups that would be transduced with AAVs expressing the Cre recombinase transgene and miR-NC or miR-Plin3. Mice were transduced with AAV2/8 vectors at the age of 6 weeks via tail vein injection of 1E+12 viral genome (V.G.) and put on 60kcal% HFD (Research Diets #D12492) after 5 days (*n* = 5 per group). Body weight was monitored weekly for up to 6 weeks. For terminal procedures, mice were fasted for 6 h (3 am–9 am), and serum, liver, and adipose tissues were collected and stored in a −80°C fridge after snap freezing. Wild-type C57BL/6J female mice were purchased from Enzwell. They were age-matched to female CGI-58^flox/flox^ mice and fed a regular chow diet. At the age of 6 weeks, these mice were transduced AAVs as described above and continued on the regular chow diet for 5 weeks. The mice were fasted for 6 h (10 am–4 pm) prior to necropsy, and liver tissues were dissected for histological studies.

### AAVs

Based on the nucleic acid sequence of mouse *Plin3* (NM_025836.3) gene, six pre-miRNA sequences per gene, and a negative control (NC) sequence was synthesized and cloned into pAAV2-CMV_bGI-MasterRNAi155-EGFP-WPRE-pA (Taitool Bioscience). *Plin3* transcripts were cloned as pAAV2-CMV_bGl-*Plin3*-mCherry-3×Flag-WPRE-pA. In a HEK293 cell system that coexpresses the miRNA and *Plin3* constructs, pre-miRNA sequences were screened via reduced fluorescence intensity of mCherry-tagged PLIN3 and reduced western blotting signals of FLAG-tagged PLIN3 proteins. The most effective sequences, i.e. miR-Plin3 (TGGTATCTAGCTCAGTGTCTA) and miR-NC (GTCTCCACGCGCAGTACATTT), were ligated into the linearization vector pAAV2-hTBG-MasterRNAi155(MCS)-Cre-GFP-WPRE-pA (Taitool Bioscience). All of the above AAVs were packaged into the AAV8 vectors and purified with titers ≥ 1.3E+13 V.G./ml by Taitool Bioscience Co.

### Metabolic phenotyping

For GTT, after 4 weeks of HFD, mice were fasted for 8 h from 9 am to 5 pm and i.p. injected with 20% glucose (2 g/kg body weight, MACKLIN #G6172). Blood glucose was measured by a glucose meter (Contour TS #1816) at 0, 15, 30, 45, 60, 90, and 120 min after glucose injection. For ITT, after 5 weeks of HFD, mice were fasted for 6 h from 9 am to 3 pm and i.p. injected with recombinant human insulin solution (0.5 IU/kg for males and 0.4 IU/kg for females, Procell #PB180432). Blood glucose was measured by a glucose meter at 0, 15, 30, 45, 60, 90, and 120 min after insulin injection. The area of the curve was generated by measuring the area under the curve and subtracting the area under the starting glucose value.

### Histology and blood chemistry tests

Mouse tissues were fixed in the 4% paraformaldehyde (Boster #AR1068). Fixed mouse liver tissues were subjected to H&E staining and PAS staining in the institution's core facility. Images were taken in an upright light microscope. Liver pathologies were scored using H&E sections according to the following criteria: hepatic steatosis (area of steatosis <5%, 0 point; 5%–33%, 1 point; 33%–66%, 2 points; >66%, 3 points) and intralobular/portal inflammation (none, 0 point; <2 spots, 1 point; 2–4 spots, 2 points; >4 spots, 3 points). No signs of ballooning degeneration were observed. Five images per H&E section were analyzed. To measure the size of adipocytes in pgWAT, 10 fields of H&E sections were randomly selected from each slide under 200× magnification and analyzed with Image Fiji (ImageJ 1.53C). Briefly, the image was calibrated to the actual scale, converted to 8-bit color, thresholded to ensure clear cell boundary, and measured for the area of each adipocyte.

Mouse sera were sampled via cardiac puncture, centrifuged for 5 min at 3000 rpm after sitting at room temperature for 20 min. Then, 100 μl sera were mixed with 400 μl of 0.9% pre-chilled saline and sent to the Laboratory Medicine Core Facility in Southwest Hospital for liver function and regular blood chemistry tests. Oil Red O staining was performed with the frozen 10-μm tissue sections following the instruction of the Oil Red reagent (Sigma #O0625).

### Liver triglyceride quantification

For analysis of the liver lipid composition, 50 mg of liver was thawed, minced, and weighed in a tube. Lipids were extracted in 2:1 chloroform/methanol at room temperature overnight. The protein was quantitatively separated from the lipid extract, which was then dried down in Vacuum drier (HENGZWELL, HZK-55) and redissolved in a measured volume of 2:1 chloroform/methanol. Diluted H_2_SO_4_ was added to the sample, which was then vortexed and centrifuged to split the phases. The aqueous upper phase was aspirated and discarded, and an aliquot of the bottom phase was removed and dried down. Next, 1% Triton X-100 in chloroform was added, and the solvent was evaporated. Then, 0.9% NaCl was added to each tube and vortexed until the solution was clear. Lipids were quantified using the Triglyceride Assay Kit (Abcam #ab65336).

### Extraction and quantification of liver contents

Tissue glycogen (10 mg tissue, Abcam #ab65620) and cholesterol (20 mg tissue, Abcam #ab65390) were measured according to the manufacturer's instructions. For protein extraction and quantification, 40 mg tissue samples were homogenized in 1 ml of prepared RIPA buffer with protease inhibitor (Roche #COUEDTAF-RO) and phosphatase inhibitor (Roche #PHOSS-RO) on ice. After centrifugation at 4°C, 13000× *g* for 20 min, the supernatant was aliquoted and quantified by the BCA method. For RNA/DNA extraction and quantification, 60–80 mg tissue samples were homogenized in 1 ml TRIzol reagent (Invitrogen #15596026) and extracted for RNA/DNA according to the manufacturer's instructions (Xin et al., 2021a). RNA/DNA concentration was measured in a spectrometer (Thermo, NanoDrop 2000C). Concentrations of these tissue biochemicals were normalized to wet weight of liver tissues.

### Free fatty acid profiling

Lipids were extracted using a modified version of the Bligh and Dyer's method as described previously (Lam et al., 2021). Briefly, 50 mg of liver samples were homogenized in 750 μl of 3:6:1 chloroform/methanol/MilliQ H_2_O (*v*/*v*/*v*) on a bead ruptor (OMNI). The homogenate was then incubated with gentle rotation at 1500 rpm for 1 h at 4°C. At the end of the incubation, 350 μl of deionized water and 250 μl of chloroform were added to induce phase separation. The samples were then centrifuged and the lower organic phase containing lipids was extracted into a clean tube. Lipid extraction was repeated once by adding 450 μl of chloroform to the remaining tissues in aqueous phase, and the lipid extracts were pooled into a single tube and dried in the SpeedVac under OH mode. Samples were stored at −80°C until further analysis.

Lipid profiling was conducted by LipidALL Technologies using an Agilent 1290 II UPLC coupled with Sciex QTRAP 6500 PLUS as reported previously (Lam et al., 2021). Individual species were separated using a Phenomenex Luna 3-μm silica column (internal diameter 150 × 2.0 mm) under the following conditions: mobile phase A (chloroform:methanol:ammonium hydroxide, 89.5:10:0.5) and mobile phase B (chloroform:methanol:ammonium hydroxide:water, 55:39:0.5:5.5). Free fatty acids were quantitated using d31-16:0 (Sigma-Aldrich) and d8-20:4 (Cayman Chemicals) as internal standards.

### Cell culture and Bodipy staining

Alpha mouse liver 12 (AML-12) cell line was derived from mouse hepatocytes (CD1 strain, line MT42) that carry a human TGF-α transgene. AML-12 cells were maintained in DMEM/F12 containing 10% fetal bovine serum, 10 μg/ml insulin, 5.5 μg/ml transferrin, 5 ng/ml selenium, 40 ng/ml dexamethasone, and 1% penicillin/streptomycin (Procell #CM-0602) and cultured at 37°C with 5% CO_2_. Cells were seeded into 12-well plates at a density of 7.5E+4 cells per well. After 12 h, cells were transfected by a mix of 50 nM Plin3 siRNA (Tsingke Biotechnology; targeting sequence: GGCTCAAGAAATGGTATCT) or control siRNA (sense: UUCUCCGAACGUGUCACGUTT; anti-sense: ACGUGACACGUUCGGAGAATT) and Lipofectamine 3000 (Invitrogen #L3000008). After 36 h of RNA interference, cells were treated with 200 μM oleic acid (Sigma #O1008) and DMSO vehicle (Boster #PYG0040) for 12 h. Cells were extracted for RNA (*n* = 3 wells, repeated twice), protein (*n* = 3 wells, repeated once), and Bodipy staining (*n* = 3 wells, repeated once). Results were reproducible. For Bodipy staining, cells were fixed with 4% paraformaldehyde for 15 min, stained with 10 nM Bodipy 493/503 solution (Invitrogen #D3922) for 10 min, and then stained with DAPI (Beyotime #C1005) for 5 min. Cells were imaged with a fluorescence microscope (Nikon Ni-U).

### Real-time quantitative polymerase chain reaction (RT-qPCR)

Liver tissue RNA was extracted by the Eastep Super Total RNA Extraction Kit (Promega #LS1040). AML-12 cell RNA was extracted by TRIzol (Invitrogen #15596026) with 500 μl reagent per well of a 12-well plate. A total of 1000 ng total RNA was reversely transcribed into complementary DNA using the GoScript Reverse Transcription Mix, Random Primers (Promega). RT-qPCR reactions were set up in a 10-μl volume (equivalent of 10 ng total RNA) using iTaq Universal SYBR Green Supermix (Bio-Rad #1725124) and a PCR thermal cycler (Bio-Rad C1000). Primer sequences are listed in Supplementary Table S1. The program was 95°C for 3 min, followed by 40 cycles of 95°C for 10 sec and 60°C for 30 sec. Data were quantified by the standard curve method as described (Xin et al., 2021b).

### Western blotting

Tissue or cell samples were lysed in RIPA buffer supplemented with proteinase inhibitors and protein phosphatase inhibitors. A total of 30 μg tissue protein or 20 μg cell protein was separated by Sodium dodecyl sulfate–polyacrylamide gel electrophoresis at 80 V and transferred to the activated polyvinylidene fluoride (PVDF) membranes (Bio-Rad #1620177). The membranes were blocked in 5% skim milk or bovine serum albumin solution for 1 h and incubated with primary antibodies at 4°C overnight. Western blotting was visualized by peroxidase-conjugated secondary antibodies and ECL chemiluminescent substrate (Bio-Rad #1705062) in the Azure C500 imaging system. When required, the PVDF membranes were stripped in antibody removal solution (Beyotime #P0025B) for 20 min and washed three times in Tris-buffered saline with 0.05% Tween 20 before re-starting the blocking and western blotting procedure. Experiments were repeated at least twice.

Cleaved caspase 3 (#9664S), RIPK3 (#95702S), OGT (#24083), β-Tubulin (#2128S), HSP60 (#12165), HSP70 (#46477), and HRP-conjugated anti-rabbit IgG (#7074) or anti-mouse IgG (#7076) antibodies were purchased from Cell Signaling Technology. PLIN2 (#15294-1-AP), PLIN3 (#10694-1-AP), and PLIN5 (#26951-1-AP) antibodies were purchased from ProteinTech. CGI-58 (#NB110-41576) antibody was from Novus Inc. p-MLKL (#ab196436) antibody was from Abcam. ATGL (#A5126), HSL (#A15686), p-HSL S563 (#AP0851), and p-HSL S660 (#AP0853) antibodies were from Abclonal.

### Statistical analysis

Data were presented as mean ± SEM. Data analysis and illustration were performed in Prism (GraphPad, v8.0.1). Two-way analysis of variance (ANOVA) post-hoc Bonferroni tests or unpaired *t*-tests were performed. *P *< 0.05 reaches statistical significance. Raw data for immunoblots and imaging studies are deposited in Mendeley Data, doi: 10.17632/wrvfd2wdwf.1.

## Supplementary Material

mjac055_Supplemental_File

## Notes

**Author contributions:** conceptualization by L.Y., M.-D.L., and Z.Z.; methodology by X.B., R.H., J.C., H.X., F.D., L.Y., and M.-D.L.; investigation by X.B., R.H., J.C., H.X., X.M., M.Z., M.-D.L., and Z.Z.; formal analysis, data curation, and visualization by X.B. and Z.Z.; original draft writing by X.B., M.-D.L., and Z.Z.; revision and editing by all authors; resources by F.D., Q.Z., G.S., and Z.Z.; supervision by S.T., M.-D.L. and Z.Z.; project administration by M.-D.L., and Z.Z.; funding acquisition by Z.Z.
