# Supplementary material for: Knockdown of hepatocyte Perilipin-3 mitigates hepatic steatosis and steatohepatitis caused by hepatocyte CGI-58 deletion in mice
Source: J Mol Cell Biol. 2022 Sep 15;14(8):mjac055. doi: 10.1093/jmcb/mjac055 (PMC9929509; doi:10.1093/jmcb/mjac055)
Supplement: mjac055_Supplemental_File [file mjac055_supplemental_file.pdf]

Supplementary Figures

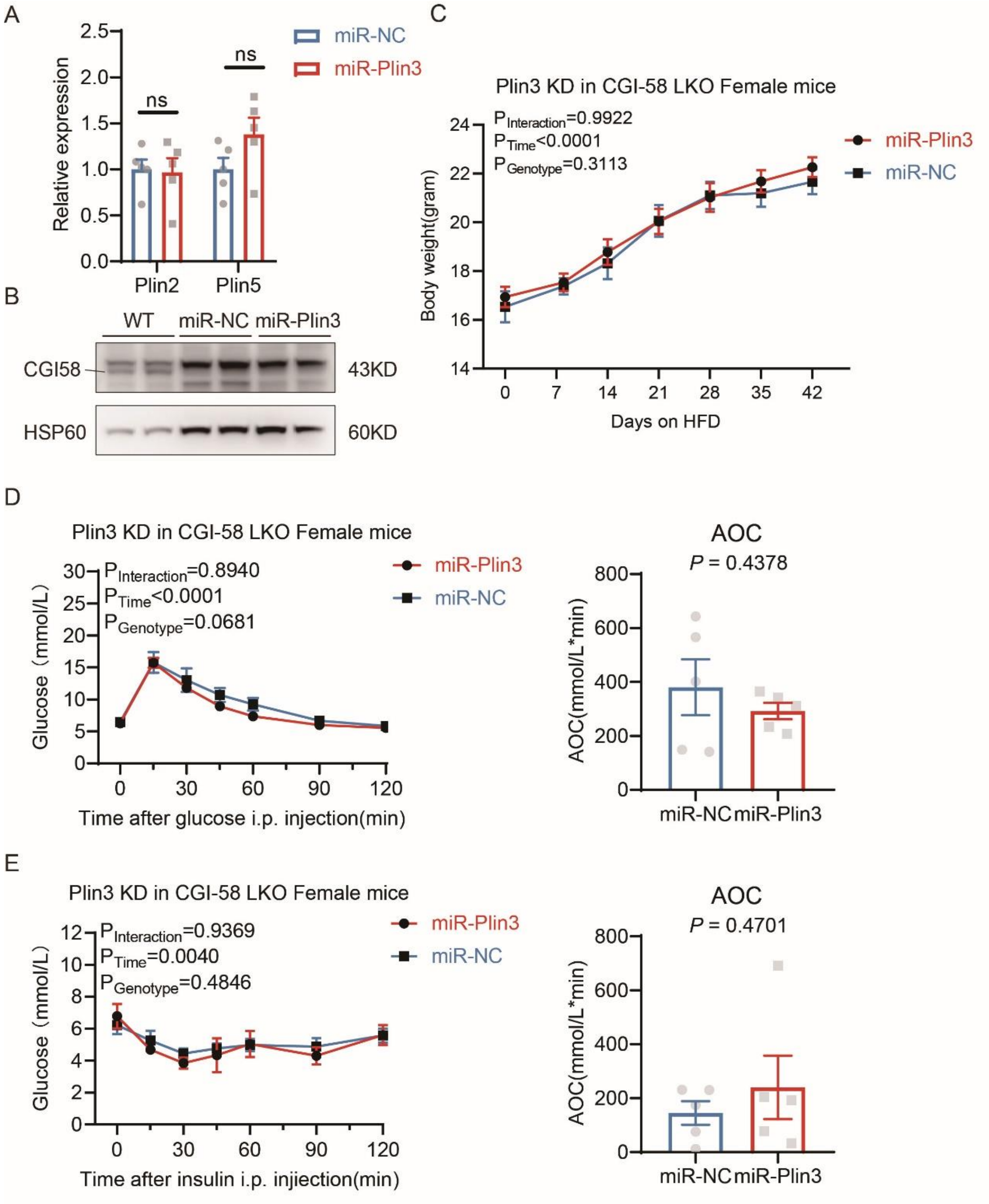

Supplementary Figure S1 Expression profile of major PAT family genes in hepatocyte-specific CGI-58 knockout mice. (**A**) RT-qPCR analysis of *Plin2/5* in miR-Plin3 CGI-58 deficient livers from female mice (n = 5). (**B**) Proteins levels of CGI-58 in mouse livers. WT, wildtype; miR-NC, control miRNA; miR-Plin3, CGI-58 deficient mice transduced with miR-Plin3. (**C**) Body weight in CGI-58^{Dliver} mice transduced with miR-Plin3 and miR-NC (n = 5). miR-NC mice were littermates of miR-Plin3 mice. (**D**) Glucose tolerance test. (**E**) Insulin tolerance test. Data were presented as mean ± SEM, unpaired t-test or two-way ANOVA, *P < 0.05.

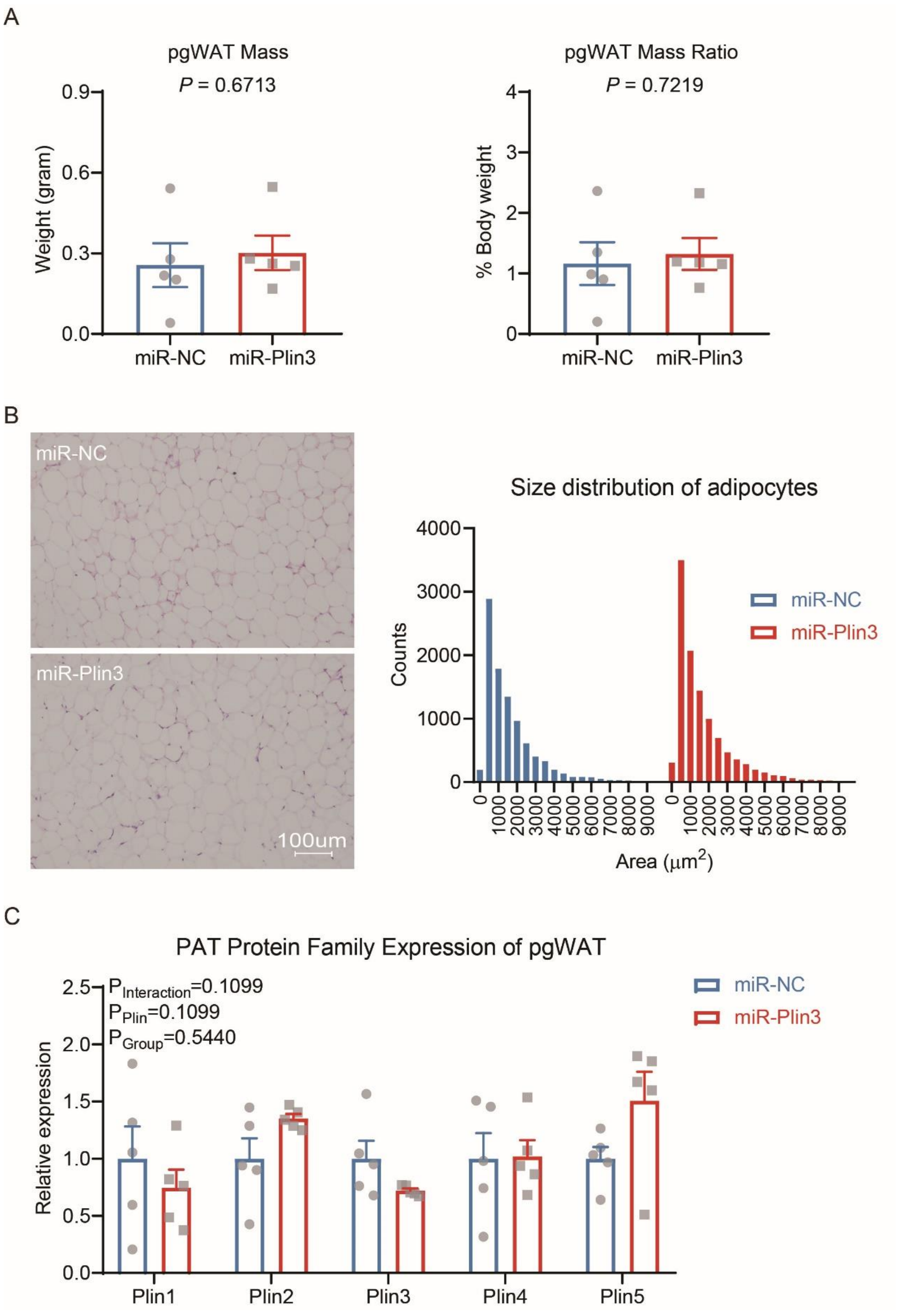

Supplementary Figure S2 Effect on adipose tissue in hepatocyte-specific CGI-58 knockout mice. (**A**) The mass and mass ratio of perigonadal white adipose tissue (pgWAT) in CGI-58$^{\Delta liver}$ mice transduced with miR-Plin3 or miR-NC (*n* = 5). (**B**) Size distribution of adipocytes. Scale: 100 μm. (**C**) Gene expression in pgWAT. Data were presented as mean ± SEM, unpaired *t*-test or two-way ANOVA, $^*P < 0.05$.

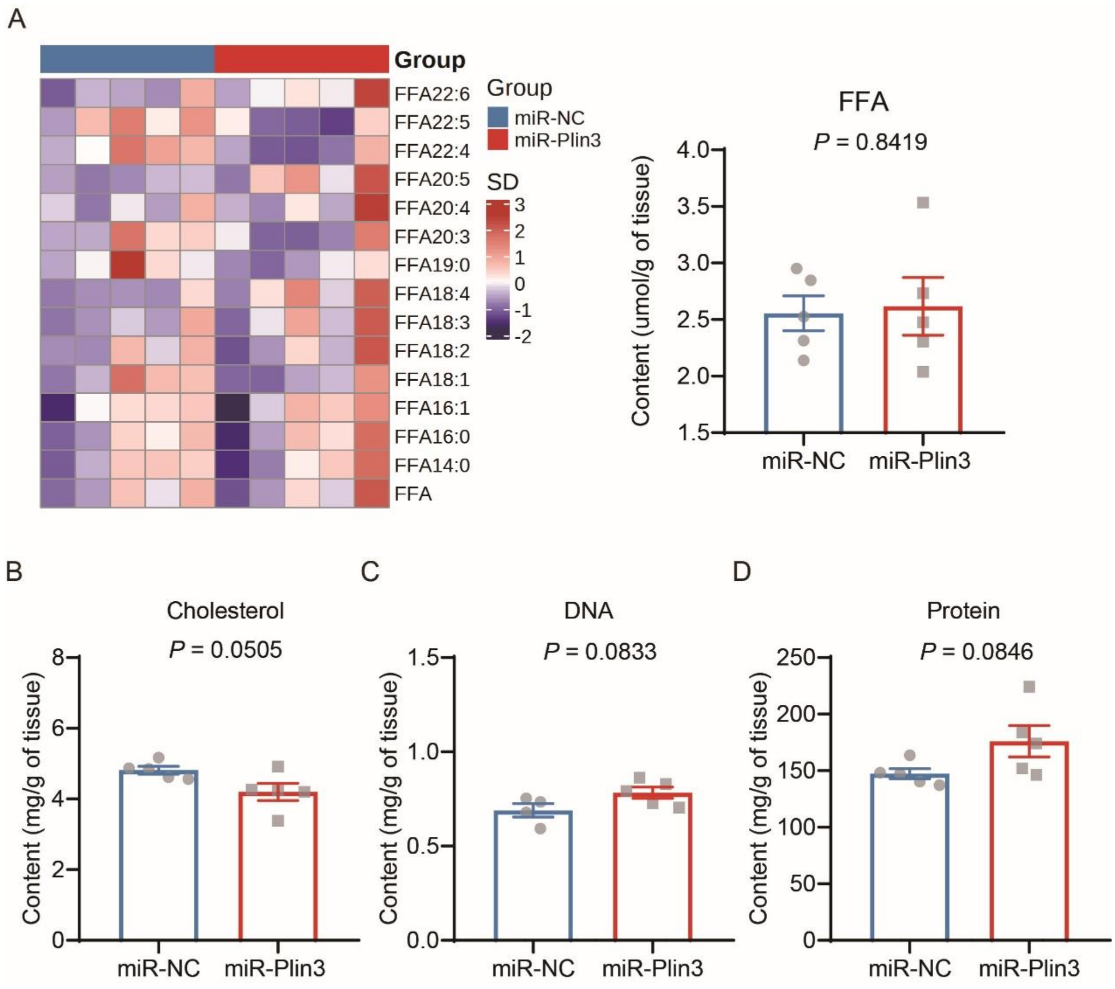

Supplementary Figure S3 Changes of macromolecular components in hepatocyte-specific CGI-58 knockout livers. Quantitative analysis of long-chain free fatty acids (FFA) by lipidomics (**A**), tissue cholesterol (**B**), tissue DNA (**C**), and tissue protein (**D**) contents in the CGI-58$^{\Delta liver}$ livers expressing miR-NC or miR-Plin3 (n = 5). Data were presented as mean ± SEM, t-test, *P < 0.05.

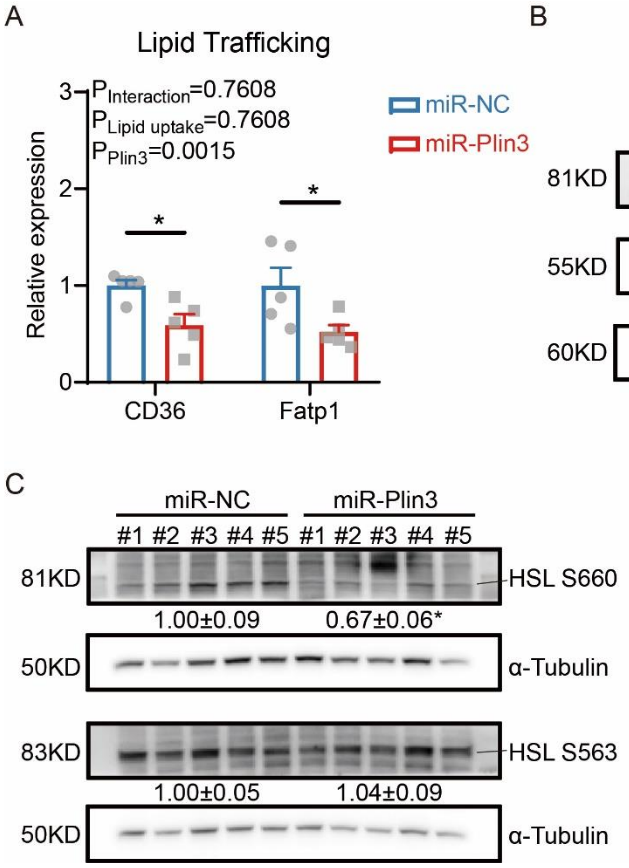

Supplementary Figure S4 Lipid uptake and lipolysis in hepatocyte-specific CGI-58 knockout livers. (**A**) Gene expression of fatty acid transporters and chaperone in the liver (*n* = 5). (**B**) Western blotting analysis of hormone-sensitive lipase (HSL) and adipose triglyceride lipase (ATGL) in mouse livers (*n* = 5). (**C**) Western blotting analysis of HSL phosphorylation in mouse livers (*n* = 5). Data were presented as mean ± SEM, unpaired *t*-test.

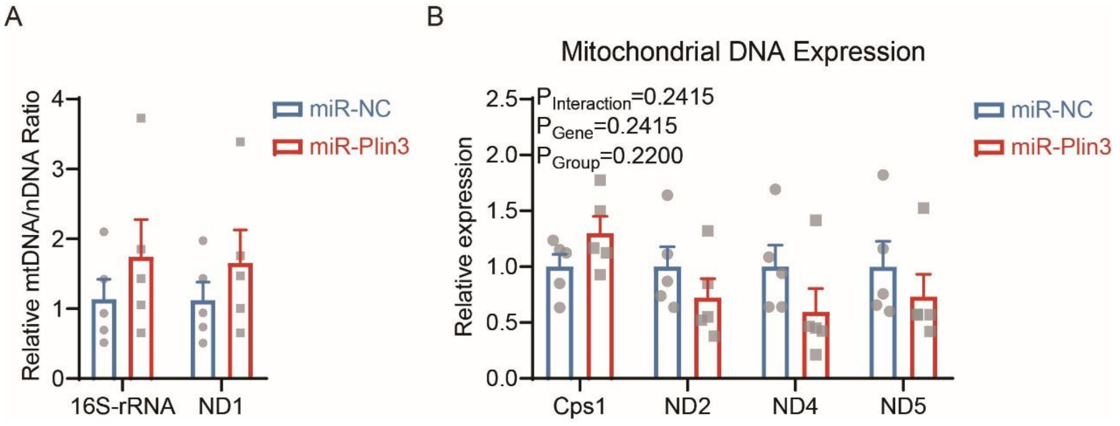

Supplementary Figure S5 Mitochondrial biogenesis was not altered by *Plin3* knockdown in hepatocyte-specific CGI-58 knockout livers. (**A**) Mitochondrial copy number (*n* = 5). Signals of mitochondrial DNA were normalized to signals of the nuclear DNA (the *Hk2* gene). (**B**) Transcript levels of *Cps1* and mitochondrial DNA-encoded genes (*n* = 5). Data were presented as mean ± SEM, unpaired *t*-test or two-way ANOVA, $*P < 0.05$.

Supplementary Tables

Supplementary Table S1 Primer sequences used in RT–qPCR analysis.

#	Gene	Name	Oligo sequence
1	Plin1	Plin1-F	CAAGCACCTCTGACAAGGTTC
2		Plin1-R	GTTGGCGGCATATTCTGCTG
1	Plin2	Plin2-F	GGAGGAAAGACTGCCTATTCTGA
2		Plin2-R	CATCCTTCGCCCCAGTTACG
3	Plin3	plin3-F	ATGTCTAGCAATGGTACAGATGC
4		plin3-R	CGTGGAACTGATAAGAGGCAGG
5	Plin4	Plin4-F	CGGCCCTTGTCGGAACTAAG
6		Plin4-R	CTTTGAAGTGTCAAGACCTCCC
7	Plin5	Plin5-F	TGTCCAGTGCTTACAACTCGG
8		Plin5-R	CAGGGCACAGGTAGTCACAC
9	CGI58	CGI58-F	TGGTGTCCCACATCTACATCA
10		CGI58-R	CAGCGTCCATATTCTGTTTCCA
11	CD68	Cd68-F	GGACCCACAACTGTCACTCAT
12		Cd68-R	AAGCCCCACTTTAGCTTTACC
13	Ccr2	Ccr2-F	ATCCACGGCATACTATCAACATC
14		Ccr2-R	CAAGGCTCACCATCATCGTAG
15	Ccl2	Ccl2-F	TTAAAAACCTGGATCGGAACCAA
16		Ccl2-F	GCATTAGCTTCAGATTTACGGGT
17	TNFα	TNFα-F	CTGGATGTCAATCAACAATGGGA
18		TNFα-R	ACTAGGGTGTGAGTGTTTTCTGT
19	Il6	Il6-F	TAGTCCTTCCTACCCCAATTTCC
20		Il6-R	TTGGTCCTTAGCCACTCCTTC
21	Il1b	Il1b-F	GAAATGCCACCTTTTGACAGTG
22		Il1b-R	TGGATGCTCTCATCAGGACAG
23	Il18	Il18-F	GACTCTTGCGTCAACTTCAAGG
24		Il18-R	CAGGCTGTCTTTTGTCAACGA
25	Nlrp3	Nlrp3-F	ATTACCCGCCCGAGAAAGG
26		Nlrp3-R	TCGCAGCAAAGATCCACACAG
27	Icam1	Icam1-F	GTGATGCTCAGGTATCCATCCA
28		Icam1-R	CACAGTTCTCAAAGCACAGCG
29	Mmp2	Mmp2-F	CCTGGACCCTGAAACCGTG
30		Mmp2-R	TCCCCATCATGGATTCGAGAA
31	Col1a1	Col1a1-F	GCTCCTCTTAGGGGCCACT
32		Col1a1-R	CCACGTCTCACCATTGGGG
33	CD36	Cd36-F	ATGGGCTGTGATCGGAACTG
34		Cd36-R	GTCTTCCCAATAAGCATGTCTCC
35	Fatp1	Fatp1-F	GCTTCAACAGCCGTATCCTC
36		Fatp1-R	TCTTCTTGTTGGTGGCACTG
37	16S rRNA	16S rRNA-F1	CCGCAAGGGAAAGATGAAAGAC
38		16S rRNA-R1	TCGTTTGGTTTCGGGGTTTC

39	HK2	HK2-F1	GCCAGCCTCTCCTGATTTTAGTGT
40		HK2-R1	GGGAACACAAAAGACCTCTTCTGG
41	ND1	ND1-F2	CTAGCAGAAACAAACCGGGC
42		ND1-R2	CCGGCTGCGTATTCTACGTT
43	ND2	ND2-F	ATACTTCGTCACACAAGCAACA
44		ND2-R	GGCCTAGTTTTATGGATAGGGCT
45	ND4	ND4-F	TCCTCAGACCCCCTATCCAC
46		ND4-R	TGGTTCCCTCATCGGGTAATAA
47	ND5	ND5-F	CACTCAGACCCAAACATCAATCG
48		ND5-R	TCGTCCGTACCATCATCCAATTA
49	Cps1	Cps1-F	ACATGGTGACCAAGATTCCTCG
50		Cps1-R	TTCCTCAAAGGTGCGACCAAT
55	U36B4	u36b4-F	AGATGCAGCAGATCCGCAT
56		u36b4-R	GTTCTTGCCCATCAGCACC
57	GAPDH	GAPDH-F	AGGTCGGTGTGAACGGATTTG
58		GAPDH-R	GGGGTCGTTGATGGCAACA

Supplementary Table S2 Comparison of physiological index and blood biochemistry in hepatocyte-specific CGI-58 knockout mice.

	miR-NC	miR-Plin3	*P*-value
Physiological index			
Body weight (g)	21.64 ± 0.51	22.60 ± 0.45	0.1945
AOC of GTT (mmol/L·min)	380.40 ± 103.60	292.20 ± 30.43	0.4378
AOC of ITT (mmol/L·min)	144.70 ± 43.61	239.70 ± 117.40	0.4701
Blood biochemical parameters			
Total bilirubin (mmol/L)	5.60 ± 1.04	5.00 ± 0.89	0.6737
Direct bilirubin (mmol/L)	2.82 ± 0.62	1.84 ± 0.41	0.2269
Indirect bilirubin (mmol/L)	2.78 ± 0.62	3.16 ± 0.79	0.7147
Total protein (g/L)	63.20 ± 1.02	63.20 ± 0.86	>0.9999
Albumin (g/L)	30.30 ± 0.85	33.30 ± 0.64	0.0224*
Globulin (g/L)	32.90 ± 0.73	29.90 ± 0.29	0.0052**

Blood biochemical parameters are measured after 6h fast. Data are presented as mean ± SEM, *t*-test (*$P < 0.05$, **$P < 0.01$).
